# Differences in the fecal microbiota of neonates born at home or in the hospital

**DOI:** 10.1038/s41598-018-33995-7

**Published:** 2018-10-23

**Authors:** Joan L. Combellick, Hakdong Shin, Dongjae Shin, Yi Cai, Holly Hagan, Corey Lacher, Din L. Lin, Kathryn McCauley, Susan V. Lynch, Maria Gloria Dominguez-Bello

**Affiliations:** 1 New York University Rory Meyers College of Nursing, New York, 10010 USA; 2 Department of Food Science and Biotechnology, College of Life Science, Sejong University, Seoul, 05006 South Korea; 3 New York University School of Medicine, New York, 10016 USA; 4 Department of Biochemistry and Microbiology and Department of Anthropology, Rutgers University, New Brunswick, 08901 USA; 5 University of California San Francisco, Department of Medicine, Division of Gastroenterology, San Francisco, 94118 USA

**Keywords:** Microbiome, Risk factors

## Abstract

Research on the neonatal microbiome has been performed mostly on hospital-born infants, who often undergo multiple birth-related interventions. Both the hospital environment and interventions around the time of birth may affect the neonate microbiome. In this study, we determine the structure of the microbiota in feces from babies born in the hospital or at home, and from vaginal samples of their mothers. We included 35 vaginally-born, breast-fed neonates, 14 of whom delivered at home (4 in water), and 21 who delivered in the hospital. Feces from babies and mothers and maternal vaginal swab samples were collected at enrollment, the day of birth, followed by days 1, 2, 7, 14, 21, and 28. At the time of birth, the diversity of the vaginal microbiota of mothers delivering in the hospital was lower than in mothers delivering at home, and showed higher proportion of *Lactobacillus*. Among 20 infants not exposed to perinatal maternal antibiotics or water birth, fecal beta diversity differed significantly by birth site, with hospital-born infants having lower *Bacteroides*, *Bifidobacterium*, *Streptococcus*, and *Lactobacillus*, and higher *Clostridium* and *Enterobacteriaceae* family (LDA > 3.0), than babies born at home. At 1 month of age, feces from infants born in the hospital also induced greater pro-inflammatory gene expression (*TLR4*, *IL-8*, *occludin* and *TGFβ*) in human colon epithelial HT-29 cells. The results of this work suggest that hospitalization (perinatal interventions or the hospital environment) may affect the microbiota of the vaginal source and the initial colonization during labor and birth, with effects that could persist in the intestinal microbiota of infants 1 month after birth. More research is needed to determine specific factors that alter bacterial transmission between mother and baby and the long-term health implications of these differences for the developing infant.

## Introduction

Hospitalization for childbirth is considered a foundation of safe obstetric care, and provides access to many life-saving interventions. Yet many interventions, such as Cesarean section, are currently overused with the assumption that there are no consequences for mother or baby. Neonates are exposed to dense vaginal bacterial communities during labor and delivery, an exposure lacking in C-section born infants, who acquire skin-like microbiota^1^, likely from the operating room^2^. Antibiotics add a compounded effect to the lack of vaginal exposure of C-section born babies, and are also used extensively during the perinatal period. A growing body of evidence suggests that disruption in early microbiome development is associated with health consequences later in life that arise from immune or metabolic dysfunction^3–8^.

Routine hospital interventions during labor and delivery may include reduced maternal meals, reduced maternal mobility, IV hydration, analgesia, anesthesia, oxytocin, frequent vaginal exams or vaginal cleansing, and episiotomy, along with stressors of microbial transmission such as intrapartum antibiotics, antibiotic eye prophylaxis to the neonate, separation of mother and baby, and early infant bathing. The hospital environment provides both an antiseptic environment for labor and delivery, as well as possible exposure to antibiotic resistant bacteria^9^. How the neonatal microbiome develops in the absence of all interventions is not well known. In this study we compare the fecal microbiota from vaginally delivered neonates born at home or in the hospital. We also determine effects associated with perinatal antibiotics in the hospital, and with water birth at home.

## Results

### Subjects

A one-month prospective cohort study was conducted on 35 vaginally-delivered infants (21 born in the hospital and 14 at home), and their 34 mothers (see Supplementary Fig. S1 and Table S1). All infants were delivered with the help of midwives, had early skin-to-skin contact, and were exclusively breastfed in the first month of life. Among the 14 babies born at home, 10 were born in air and 4 in water. No babies in either group received direct antibiotic treatment in the first month of life, but among mothers delivering in the hospital, 11 mothers received antibiotic treatment: 4 received one dose of IV antibiotics for group B strep prophylaxis, and 7 received oral antibiotics perinatally (in the month preceeding or following delivery), for indications such as urinary and upper respiratory infections. (See Supplementary Table S1).

### Infants born at home or in the hospital

Fecal 16S *rRNA* gene sequences from the first month (day 0, 1, 2, 7, 14, 21, and 28) of 20 vaginally-delivered breastfed infants not exposed to maternal antibiotics or water birth (10 born at home and 10 in the hospital) were used to compare infants by delivery site (see Supplementary Table S2). 16S *rRNA* gene sequences were used to identify and quantify operational taxonomic units (OTUs) with open-reference OTU picking method using 97% identity to the Greengenes database (v13_8).

**Figure 1:**
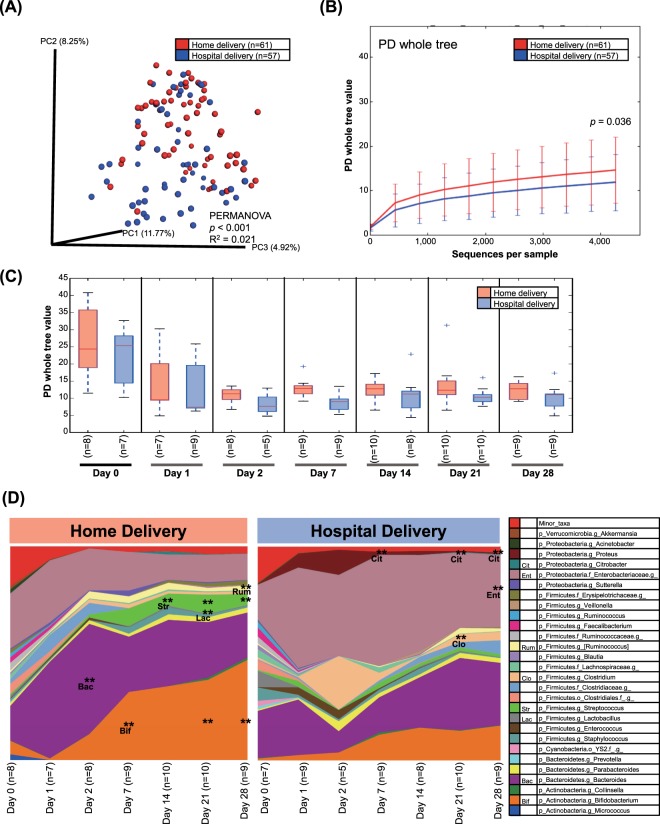
Fecal microbiota diversity in vaginally-delivered infants, 10 delivered at home and 10 in the hospital. (**A**) Fecal β-diversity. Unweighted UniFrac distances were used to evaluate β-diversity. PERMANOVA was used to test dissimilarity. (**B**) Rarefaction curves. Phylogenetic diversity (PD) was used to plot bacterial diversity. Non-parametric p value was calculated using 10,000 Monte Carlo permutations. (**C**) Fecal α-diversity stratified by days after birth. (**D**) Taxa plots. All babies were breastfed and were not exposed to antibiotics.

Hospitalization was associated with alterations in the neonatal microbiota structure during the first month (Unweighted PERMANOVA *p* < 0.001, R^2^ = 0.021; Fig. 1A), but the differences were greatest at day 2 of life (Unweighted PERMANOVA *p* = 0.018, R^2^ = 0.121; Weighted PERMANOVA *p* = 0.007, R^2^ = 0.338; see Supplementary Fig. S2). Differences included marginally lower fecal alpha diversity in infants born in the hospital relative to home-born neonates (Phylogenetic diversity, *p* = 0.036; Fig. 1B,C). Discriminant taxa by site of delivery (based on Linear Discrimination Analysis, LDA > 3.0 of bacterial OTUs in proportions >1%), included lower proportions of *Bacteroides* (day 2), *Bifidobacterium* (day 7, 21, 28), *Streptococcus* (day 14, 21, 28), *Lactobacillus* (day 21) in hospital-born infants (Fig. 1D and Supplementary Fig. S3). After the third week of life, hospital-born infants had higher proportions of *Clostridium* (day 21), *Enterobacteriaceae* (day 28) and *Citrobacter* (day 21, 28) than home-born infants (Fig. 1D and Supplementary Fig. S3).

### Infants exposed to maternal peripartum antibiotics

When considering the effects of perinatal maternal antibiotics (see Supplementary Table S3), there were no significant differences in the fecal microbiota between the hospital-born babies with or without peripartum antibiotics, in either beta (see Supplementary Figs S4 and S5) or alpha diversity (see Supplementary Fig. S6).

### Infants born in water

When comparing the effects of water birth in 4 of the 14 home-born infants, infants born in water showed beta diversity differences in their fecal microbiota. Water-born infants showed higher relative abundances of *Erysipelotrichaceae* and *Comamonadaceae* families in relation to air-born, despite no differences in alpha diversity (see Supplementary Fig. S7). However, the small sample size resulted in insufficient statistical power for comparisons (see Supplementary Table S4 and Fig. S7).

### Mothers delivering at home or in the hospital

**Figure 2:**
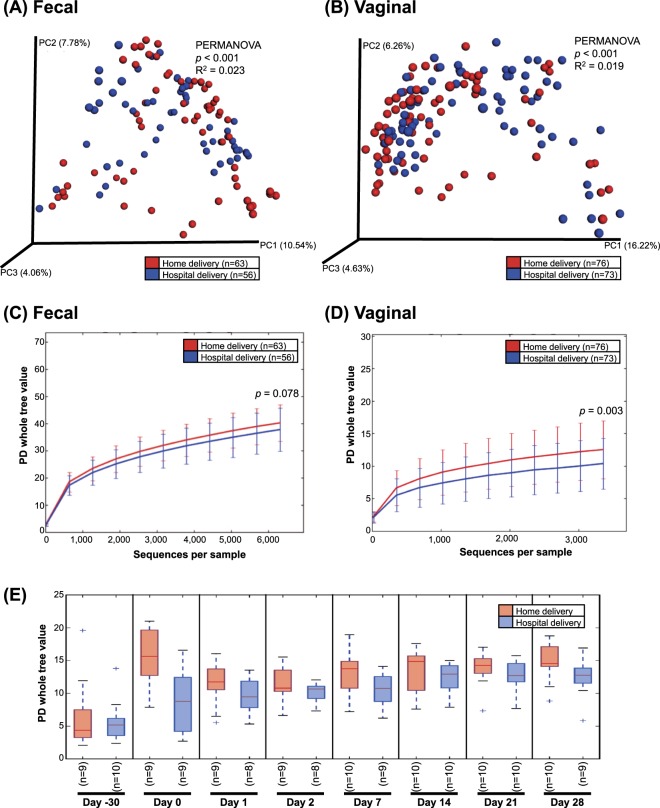
Diversity of the maternal fecal and vaginal microbiotas in mothers, 10 delivering at home and 10 in the hospital. (**A**,**B**) β-diversity of fecal microbiota (**A**) and vaginal microbiota (**B**). Unweighted UniFrac distances were used to evaluate β-diversity. PERMANOVA was used to test dissimilarity. (**C**,**D**) α-diversity of fecal microbiota (**C**) and vaginal microbiota (**D**). Phylogenetic diversity was used to plot bacterial diversity. Non-parametric p value was calculated using 10,000 Monte Carlo permutations. (**E**) Vaginal α-diversity stratified by days after birth.

The maternal fecal microbiota differed significantly between those delivering at home or in the hospital without antibiotic exposure (Unweighted; PERMANOVA, *p* < 0.001, R^2^ = 0.023; Fig. 2 and Supplementary Fig. S8), and no differences in alpha diversity were noted (see Supplementary Fig. S9). While dyads of mothers and their babies tended to show close distances in Procrustes analysis, the statistical power was low to make comparisons (see Supplementary Fig. S10).

At the time of birth, maternal vaginal beta diversity was significantly different between the mothers in the two groups (Unweighted; PERMANOVA, *p* < 0.001, R^2^ = 0.019; Fig. 2B and Supplementary Fig. S11), with hospital-delivering mothers having lower alpha diversity (Fig. 2C–E and Supplementary Fig. S12), lower proportions of *Corynebacterium*, *Dialister*, *Veillonella*, *Finegoldia*, and *Peptoniphilus* (LDA > 3.0) and higher proportion of *Lactobacillus* in relation to mothers delivering at home (LDA > 3.0; see Supplementary Fig. S13). Regardless of delivery location, maternal vaginal alpha diversity was increased during the first month after delivery (Fig. 2E and Supplementary Fig. S12).

### Degree of pro-inflammatory gene expression in epithelial cells by the fecal microbiota of infants

**Figure 3:**
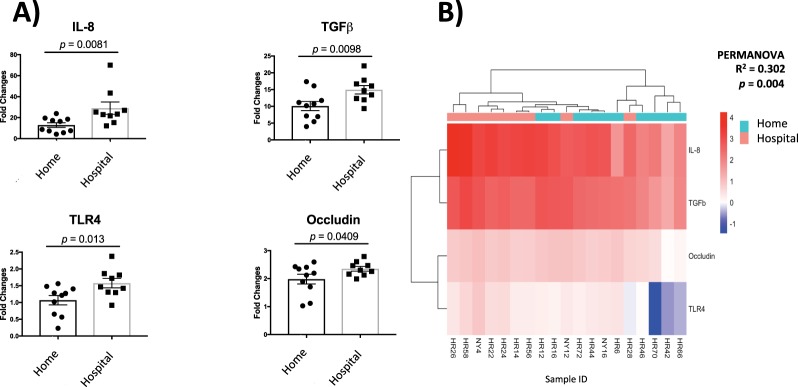
Human colonic epithelial HT-29 cells gene expression following exposure to sterile fecal water generated from 1 month old infant feces delivered in a home (n = 10) or hospital environment (n = 9). (**A**) Gene expression of a subset of epithelial markers differs by home or hospital birth. Mean ± SEM are shown. (**B**) Unsupervised heatmap plot of four epithelial genes indicates significant differences in epithelial gene expression following stimulation by the products of the gut microbiome of 1 month old infants who were delivered in a home or hospital environment.

To determine whether these differences in early life microbiota composition based on home or hospital delivery led to differences in epithelial immunostimulation by the gut microbiota at 1 month of age, human colonic epithelial HT-29 cells were exposed to sterile fecal water from babies in each of these groups and immune gene expression was profiled. A range of genes, including epithelial inflammatory cytokines IL-8, TNFα, TGFβ, microbial product sensing toll-like receptor TLR4, leukocyte adhesion mediating molecule ICAM-1, the tight junction-associated, occludin, and the mucin-producing gene, MUC2 were profiled. Of these, gene expression levels of TLR4, IL-8, occludin, and TGFβ were significantly different between home and hospital-delivered 1-month old babies (Fig. 3 and see Supplementary Fig. S14). Correlation analysis to examine relationships between relative abundance of bacterial taxa and immune reactivity showed that one *Bacteroides* genus (possibly *Bacteroides vulgatus*) was positively correlated with expressions of TGFβ and occludin (Spearman, non-parametric t-test *p* < 0.005). Home-delivered neonates exhibited significantly lower expression of all of these markers. Thus, at 1 month of age, the gut microbiome of home- and hospital-delivered babies exhibit a distinct capacity to induce epithelial gene expression, with hospital-delivered infants inducing a greater degree of pro-inflammatory gene expression.

## Discussion

Fecal microbiota differences in hospital-delivered infants, in relation to those born at home, resemble some of the reported effects of other stressors such as C-section^10,11^, antibiotics^12–14^, or formula feeding^11,12,15,16^. Namely, decreased *Bacteroides*, *Bifidobacterium* and *Ruminoccocus*, and increased *Enterobacteriaceae* and *Clostridium* species were noted. Previous studies have reported increased risk of asthma in children who had high *Clostridium* colonization in early life^17^. Further, a lower level of *Clostridium* colonization in home-delivered infants^18^ was previously associated with reduced eczema and asthma risks at age 6–7 years. Neonatal differences in the gut microbiota of home- or hospital-delivered babies also translate into differential capacity of intestinal metabolite responses. The consistency of enhanced inflammatory gene expression response and alteration of expression of genes involved in epithelial permeability, highlights the physiological relevance of even “small stressors” and differences in the very early life microbiota, during a critical developmental window. Whether hospital-delivered infants indeed show higher inflammation *in vivo*, remains unknown. If they do, there might be a mechanistic link with the increased risk that C-section birthing poses for development of immune dysfunctions later in childhood. Nonetheless, our results suggest that the delivery environment and co-associated exposures may promote durable effects on the gut microbiome’s interaction with epithelial immunity. Some microbial metabolic products are biologically active molecules, e.g. microbial linoleic acid metabolite 12, 13-diHOME^19^, propionate^20^. Microbial antigens interact with TLR receptors, eliciting robust innate and adaptive immune and endocrine responses.

A strength of this study is the homogeneity of the population in the home and hospital birth groups, both geographically (the Hudson Valley region of New York State) and racially (primarily Caucasian). Home and hospital cohorts were also well matched in terms of medical, lifestyle, and nutritional characteristics, as well as the low-intervention style of care they received from midwives. They were also well matched on labor characteristics including similar mean lengths of labor, rupture of membranes, and time to first breastfeeding. Limitations of the study include the small size and possible confounding factors such as number of siblings, use of probiotics or bactericidal soap, and rural residence. The home environment differs from the more antiseptic environment of the hospital^21^, and at home the neonate may have earlier and longer exposures to siblings, adults and pets. In our study, home birth households had a higher number of siblings at home and were more frequently located in peri-urban or rural environments, which could represent confounding factors. Previous studies have shown that infants with siblings were more likely to have a *Bifidobacterium*-dominant gut microbiota profile in relation to those without siblings^22^.

We did not note a difference between babies with or without exposure to maternal antibiotic treatment. However, the number of women receiving antibiotics was small. Due to small sample size, women receiving any antibiotic treatment in the month before, during or after labor were analyzed together which included treatments occurring at different times (pre-, intra-, and post labor) and routes (oral and IV). Antibiotics may alter the maternal microbiota to which the baby is exposed, or may pass to the baby via breast milk after delivery^23^. These preliminary results justify the need for bigger studies that control confounding factors using multivariate analyses.

The fact that not only the infant but the maternal microbiota differed by place of birth, suggests common factors exerting prenatal microbiota effects, and further, that the infant microbiota differences might be mediated by differences in the source—the maternal microbiota. We found alpha diversity of the vaginal microbiota was generally higher for the entire month after delivery compared to the month before, which is consistent with the reported decrease in vaginal alpha diversity during pregnancy^24^. There were strong group differences in the maternal vaginal microbiota at day 0, which may be due to hospital interventions, including for example, more frequent vaginal exams with lubrication (not recorded in our study) or the common use of sterile drapes and equipment in the delivery room. Routine common hospital interventions that may also perturb natural microbiota transmission, include postnatal ophthalmic antibiotics, early infant bathing with soap, separation of mother and baby to complete newborn assessments on near-sterile baby warmers, maternal betadine vaginal washes or antibiotics. The compounded effects of seemingly minor interventions may lead to major alterations in the neonatal microbiota of neonates. The results of this work suggest that hospitalization (perinatal interventions or the hospital environment) may affect the source microbiota or microbial transmission during labor and birth, with effects that could persist in the intestinal microbiota of infants 1 month after birth. This study did not include prenatal samples that would have provided a base line for the trajectory of postnatal changes in the maternal vagina and baby intestine, but differences in postnatal microbiota dynamics exist between the hopspitalized and home mothers and babies. It cannot be discarded, however, that the differences are due to confounders such as differential use of bactericidal soaps and probiotics, number of siblings and rural residence (see Supplementary Table S1). A randomized design would allow for control of the effect of these variables.

Currently around 99% of U.S. babies are delivered in hospitals^19^. Some European countries have higher proportions of home births^25^, and these are recognized as a safe alternative for low-risk women^21^. Because birth-related key microbial exposures and early microbiota perturbations are associated with later disease risks, such as atopy and asthma^19,26^, understanding the effects of hospital delivery on neonatal microbial founder populations and early-life microbiome development is critical to mitigate potential contributors to disease development. A study cohort in the Netherlands showed that full-term, breastfed babies born at home also had different intestinal microbiota than those born in the hospital, with heavier colonization of Bifidobacteria and lower colonization of *C. difficile* and *E. coli*^10^, consistent with the results of our small cohort study.

Further analysis that takes advantage of the mother-baby dyads and the longitudinal study design, such as metagenomic or metatranscriptomic analyses, will be valuable to understand differences in mechanisms and bacterial strains and functions. Also, further research is needed to determine whether differences in the infant microbiota noted here among hospital-born infants translate into *in vivo* physiological or immunological changes or clinical phenotypes. Such changes would permit insights into the benefits of home birth and permit a redesign of hospital environments and routine practices, especially those that are without strong clinical indications, to more closely resemble home conditions, thereby allowing a less perturbed physiologic and evolutionarily-refined birth process.

## Conclusion

This study shows that home versus hospital birth of exclusively breastfed, vaginally-delivered infants, is associated with differences in neonatal fecal microbiota composition and, at 1 month of age, increased capacity for hospital-delivered fecal microbiota associated products to induce pro-inflammatory epithelial gene expression. These data show that babies from mothers that opt for home delivery differ in their fecal microbiota, from babies born in the hospital, but more studies are needed to determine the specific effect that is attributable to hospital perinatal interventions.

## Methods

### Subjects and sampling

A prospective cohort study was conducted to compare breastfed vaginally-delivered infants born at home to those born in the hospital. A total of 34 mother-baby dyads were enrolled in this study, including one set of twins (34 mothers, 35 infants). Fourteen babies delivered vaginally at home and 21 delivered vaginally in the hospital. Four of the babies born at home delivered directly into water. Seven of the babies born in the hospital were exposed via maternal treatment to either intrapartum antibiotic prophylaxis (IAP) for group B strep (n = 4) or peripartum antibiotics in the month before or after delivery (n = 7) for indications such as urinary tract and upper respiratory infections (see Supplementary Table S1). There was a high consistency of practice in home and in hospital births. This included deliveries attended by midwives, early skin-to-skin contact between mother and baby after birth, early initiation of breastfeeding, no vaginal or bowel cleansing prior to delivery, and limited use of sterile drapes, gowns and masks.

Maternal, infant, pregnancy and labor characteristics were well matched between cohorts including maternal ethnicity, age, education, pre-pregnant BMI, length of labor, duration of rupture of membranes, baby weight, baby gender, and timing of breastfeeding initiation. Women in the study were healthy and without significant complications during pregnancy including gestational diabetes. Lifestyle characteristics such as exercise and diet were also well matched, and there were no smokers in the study. Labor interventions varied little between home and hospital groups.

All mothers were enrolled during the last month of pregnancy. Recruitment continued from June, 2015 through June, 2016. Home births occurred in a 5-county area in the Hudson Valley Region of New York State and hospital births occurred in a community-level hospital in the same region. Study acceptance rate was 66%. Retention rate was 100%. Study participants met the following criteria: term gestation (>37 and <42 weeks), pregnancy without complications, infants without anomalies or treatment, women 21 years of age or older, women with the ability to understand and meaningfully consent to participation, and fluency in English. This study was approved by the NYU School of Medicine, Institutional Review Board (Study # i14-00377) and all methods were performed in accordance with the relevant guidelines and regulations. Participation was voluntary and included written informed consent.

A sterile, cotton-tipped swab was used for sampling babies’ feces and mothers’ feces and vaginal swabs. Mother and baby dyad samples were collected simultaneously. Sampling was performed at enrollment, the day of birth, followed by days 1, 2, 7, 14, 21, and 28. Infant fecal samples were obtained from feces in the current diaper. Infants delivered in the hospital were discharged between 48 and 60 hours post-partum. Day 0, 1, and 2 samples were collected in the hospital and the remaining samples were collected at home. The maternal vaginal microbiota samples at day 0 were collected during labor and up to two hours after delivery. Samples were kept less than one hour between sampling, and freezing.

Non-biological data consisted of a baseline questionnaire at enrollment that included demographic, medical, lifestyle and environmental information. A birth survey was completed at the time of delivery with labor, delivery, and infant characteristics. Follow-up questionnaires were completed at each of the subsequent sampling episodes to elicit information on current feeding method and changes in mother or baby health status. This information was either directly observed, elicited from the mother and/or confirmed in the medical record.

### DNA isolation and analysis

We used approximately 5 grams of the feces collected from the diapers with sterile cotton tipped swabs and placed in sterile tubes. Samples were frozen within 1 hour of collection at <−18 °C until being transported to the lab for storage at −80 °C until processing. Total DNA was extracted from fecal samples using the Mobio Powersoil kit (MO BIO Laboratories, CA, USA), and the V4 region of the 16S *rRNA* gene was amplified with 515 F/806R V4 primers. The reverse primer contained the 3′ Illumina adapter, a unique 12-base error-correcting Golay barcode, a reverse primer pad, a two-base linker sequence (‘CC’) and the 806R primer for library construction. PCR amplification was carried out (in triplicate) using the Bio-Rad CFX 96 thermal cycler (Bio-Rad, CA, USA). Replicate amplicons were pooled, and DNA concentrations were determined using the Quant-iT PicoGreen dsDNA kit (Invitrogen, NY, USA). Amplicons were pooled in equimolar ratios and purified using QIA quick PCR purification kit (Qiagen Inc, CA, USA) and sequenced using Illumina MiSeq platform (Illumina, CA, USA) with paired-end technique. Sample processing, sequencing, and core amplicon data analysis were performed by the Earth Microbiome Project (www.earthmicrobiome.org), and all amplicon sequence data and metadata have been made public through the EMP data portal (qiita.microbio.me/emp; Study ID: 11575)^27^.

### Microbial 16S rRNA sequencing and analyses

Fecal samples from the first month of life of 20 infants (not exposed to antibiotics or water birth; 10 born at home and 10 in the hospital), generated over 10 million sequences, with an average of 25,921 sequences per sample. Sequences were analyzed using the QIIME (v.1.9.1) pipeline^28^ based on an open-reference method that maps sequences with 97% identity to known sequences in the Greengenes database (v13_8)^29^ using UCLUST^30^ and PyNAST^31^ alignment algorithms. We used Usearch61 to identify chimeric sequences and performed permutational multivariate analysis of variance (PERMANOVA) to determine differences in beta diversity. To test for statistical significance, we used non-parametric t-tests with 10,000 Monte Carlo permutations in QIIME. The linear discriminant analysis (LDA) effect size (LEfSe) algorithm^32^ was used to identify significant differences OTU relative abundance. Spearman correlations were calculated using 10,000 permutations for bootstrapped *p*-value.

### Infant sterile fecal water cell assay

The induction of inflammatory response of sterile fecal water was assayed using epithelial cells. This assay allows the determination of epithelial cell response to fecal metabolites^33^. Analysis of induction of epithelial gene expression, was performed on samples that had enough material remaining (>0.2 grams), which were those from 9 hospital and 10 home-born neonates. A doubled volume of pre-warmed 20% fetal bovine serum (FBS) in [Cat. No. 35-011-CV, CORNING, Manassas, VA 20109] PBS/4 mM EDTA was used to re-suspend stool (range 0.21–0.52 g) from swabs, suspensions were vortexed for 1–3 minutes to fully resuspend all stool material, incubated at 37 °C for 30 minutes, vortexed for 1–3 minutes, incubated at 4 °C overnight, centrifuged at 14,000 rcf (relative centrifugal force) for 10 minutes at room temperature, and the supernatant transferred to a sterile 2 mL tube. Samples were then centrifuged at 16,000 rcf for 30 minutes to remove solids before filtration of the supernatant through Whatman^TM^ 0.45 um PVDF Mini-UniPrep filters (Cat. No. US203NPEAQU, GE Healthcare Life Sciences) followed by filtration through 0.22 um filters (Cat. No. US203NPUAQU) to produce “sterile fecal water” (SFW) extracts for cell assays. Human colon epithelial HT-29 cells were seeded at a confluent density in 96-well plates in fresh McCoy’s 5A media (Cat. No. 16600-082; Thermo Fisher Scientific) supplemented with 10% heat-inactivated FCS (Cat. No. 9871-5244, USA Scientific), 100 U/ml penicillin-streptomycin (Cat. No. 10378016, Life Technologies, Carlsbad, CA) the day before treatment. After 24 hours, spent culture media was exchanged for fresh media containing SFW (final SFW concentration 25%) for 24 hours. Negative controls included media and SFW buffer. Total RNA was isolated with RNAqueous^®^-Micro Kit (Cat. No. AM1931, Thermo Fisher Scientific) and digested with DNase I. First-strand cDNA synthesis was performed using 300 ng RNA with High-Capacity RNA-to-cDNA^TM^ Kit (Cat. No. 4387406, Thermo Fisher Scientific) according to the manufacturer’s instructions.

### Real-time quantitative PCR

Real-time PCR was performed in triplicate with SYBR Green master mix (Cat. No. 4368577, Life Technologies) in the QuantStudio™ 6 Flex Real-Time PCR System (Applied Biosystems, Foster, CA). The PCR reaction conditions were as follows: 50 °C for 2 min, 95 °C for 10 min (1 cycle); 95 °C for 15 s and 60 °C for 1 min (40 cycles); and a final melting curve cycle of 95 °C for 15 s, 60 °C for 1 min and 95 °C for 15 s. The relative fold change of specific genes in expression is normalized to β-actin expression by the 2^−∆∆*CT*^ (where *C*_*T*_ is threshold cycle) method^34^. The forward and reverse primers used for PCR analysis of the investigated genes and β-actin are as follows.

IL-8, 5′-ACTGAGAGTGATTGAGAGTGGAC-3′ and 5′-AACCCTCTGCACCCAGT TTTC-3′; TLR4, 5′-AAGCCGAAAGGTGATTGTTG-3′ and 5′-CTGAGCAGGGTCT TCTCCAC-3′; TGF-β, 5′-GCGTGCTAATGGTGGAAAC-3′ and 5′-CGGTGACA TCAAAAGATAACCAC-3′; Occludin, 5′-GATGAGCAGCCCCCCAAT-3′ and 5′-GGTGAAGGCACGTCCTGTGT-3′; TNFα gene, 5′-AGGCGGTGCTTGTTCCTCAG-3′ and 5′-GGCTACAGGCTTGTCACTCG-3′; ICAM-1 gene, 5′-CAGTCACCTATGGCAACGACT-3′ and 5′-CTCTGGCTTCGTCAGAATCAC3′; MUC2 gene, 5′-CTGCACCAAGACCGTCCTCATG-3′ and 5′-GCAAGGACTGAACAAAGACTCAGAC-3′; and β-actin, 5′-AAGATGACCCAGATCATGTT TGAGACC-3′ and 5′-AGCCAGTCCAGACGCAGGAT-3′. Triplicate reactions were performed per sample and the mean *C*_*T*_ used for analysis. Unpaired Student’s t-test was used for significance testing.

## Electronic supplementary material

Supplementary Material

## Data Availability

All data generated or analyzed during this study are included in this published article (and its Supplementary Materials) and made public through the EMP data portal (qiita.microbio.me/emp; Study ID: 11575).
