# Author Correction: Differences in the fecal microbiota of neonates born at home or in the hospital

**DOI:** 10.1038/s41598-019-44426-6

**Published:** 2019-06-18

**Authors:** Joan L. Combellick, Hakdong Shin, Dongjae Shin, Yi Cai, Holly Hagan, Corey Lacher, Din L. Lin, Kathryn McCauley, Susan V. Lynch, Maria Gloria Dominguez-Bello

**Affiliations:** 1 New York University Rory Meyers College of Nursing, New York, 10010 USA; 2 Department of Food Science and Biotechnology, College of Life Science, Sejong University, Seoul, 05006 South Korea; 3 New York University School of Medicine, New York, 10016 USA; 4 Department of Biochemistry and Microbiology and Department of Anthropology, Rutgers University, New Brunswick, 08901 USA; 5 University of California San Francisco, Department of Medicine, Division of Gastroenterology, San Francisco, 94118 USA

Correction to: *Scientific Reports* 10.1038/s41598-018-33995-7, published online 23 October 2018

The original version of this Article contained errors. In the abstract,

“At the time of birth, the diversity of the vaginal microbiota of mothers delivering in the hospital was higher than in mothers delivering at home, and showed higher proportion of *Lactobacillus*”.

now reads:

“At the time of birth, the diversity of the vaginal microbiota of mothers delivering in the hospital was lower than in mothers delivering at home, and showed higher proportion of *Lactobacillus*”.

Additionally in the introduction section,

“Neonates are exposed to dense vaginal bacterial communities during labor and delivery, an exposure lacking in C-section born infants, who acquire skin-like microbiota^1^, likely from the operating room^2^. Antibiotics add a compounded effect to the lack of vaginal exposure of C-section born babies, and are also used extensively during the perinatal period for all women”.

now reads:

“Neonates are exposed to dense vaginal bacterial communities during labor and delivery, an exposure lacking in C-section born infants, who acquire skin-like microbiota^1^, likely from the operating room^2^. Antibiotics add a compounded effect to the lack of vaginal exposure of C-section born babies, and are also used extensively during the perinatal period”.

Moreover in the results section, under subheading “Mothers delivering at home or in the hospital”.

“At the time of birth, maternal vaginal beta diversity was significantly different between the mothers in the two groups (Unweighted; PERMANOVA, *p* < 0.001, R^2^ = 0.019; Fig. 2B and Supplementary Fig. S11), with hospital-delivering mothers having higher alpha diversity (Fig. 2C–E and Supplementary Fig. S12), lower proportions of *Corynebacterium*, *Dialister*, *Veillonella*, *Finegoldia*, and *Peptoniphilus* (LDA > 3.0) and higher proportion of *Lactobacillus* in relation to mothers delivering at home (LDA > 3.0; see Supplementary Fig. S13). Regardless of delivery location, maternal vaginal alpha diversity was increased during the first month after delivery (see Supplementary Fig. S11)”.

now reads:

“At the time of birth, maternal vaginal beta diversity was significantly different between the mothers in the two groups (Unweighted; PERMANOVA, *p* < 0.001, R^2^ = 0.019; Fig. 2B and Supplementary Fig. S11), with hospital-delivering mothers having lower alpha diversity (Fig. 2C–E and Supplementary Fig. S12), lower proportions of *Corynebacterium*, *Dialister*, *Veillonella*, *Finegoldia*, and *Peptoniphilus* (LDA > 3.0) and higher proportion of *Lactobacillus* in relation to mothers delivering at home (LDA > 3.0; see Supplementary Fig. S13). Regardless of delivery location, maternal vaginal alpha diversity was increased during the first month after delivery (Fig. 2E and Supplementary Fig. S12)”.

Finally in the methods section, under subheading “Subjects and sampling”

“A total of 36 mother-baby dyads were enrolled in this study, including one set of twins (34 mothers, 35 infants)”.

now reads:

“A total of 34 mother-baby dyads were enrolled in this study, including one set of twins (34 mothers, 35 infants)”.

These errors have now been fixed in the HTML and the PDF versions of the original article.

