# Supplementary material for: Differences in the fecal microbiota of neonates born at home or in the hospital
Source: Sci Rep. 2018 Oct 23;8:15660. doi: 10.1038/s41598-018-33995-7 (PMC6199260; doi:10.1038/s41598-018-33995-7)
Supplement: Supplementary file 1 — Supplementary Material [file 41598_2018_33995_MOESM1_ESM.pdf]

Manuscript Title: Differences in the fecal microbiota of neonates born at home or in the hospital

Authors: Joan L Combellick[1^], Hakdong Shin[2^], Dongjae Shin[2], Yi Cai[3], Holly Hagan[1], Corey Lacher[4], Din L. Lin[5], Kathryn McCauley[5], Susan V. Lynch[5] and Maria Gloria Dominguez-Bello[3, 4*]

Affiliations:
[1] New York University Rory Meyers College of Nursing, New York, 10010, USA
[2] Department of Food Science and Biotechnology, College of Life Science, Sejong University, Seoul, 05006, South Korea
[3] New York University School of Medicine, New York, 10016, USA
[4]Department of Biochemistry and Microbiology and Department of Anthropology, Rutgers University, New Brunswick, 08901, USA
[5]University of California San Francisco, Department of Medicine, Division of Gastroenterology, San Francisco, 94118, USA

^, These authors contributed equally to this work

Corresponding author:
Maria Gloria Dominguez-Bello
Email: mg.dominguez-bello@rutgers.edu

Supplementary Material

Supplementary Information:
Supplementary Tables 1-4 and Supplementary Figures S1-14

Table S1. Demographic and general characteristics of the subjects in the study

MATERNAL CHARACTERISTICS	HOME BIRTH n=14 mom/14 baby	HOSPITAL BIRTH n=20 mom/21 baby
Age Mean (min-max) SD	32.43 (26 – 42) 4.54	32.85 (22 – 41) 4.96
Pre-pregnant BMI Mean (min-max) SD	23.05 (19.7-34.4) 3.55	23.89 (19.6 – 29.9) 3.01
Education: ≥some college (%)	100	95
Caucasian (%)	92.9	80
Multiparity (%)	92.9	50
Maternal Smoking (%)	0	0
Maternal Exercise: ≥2-3 X per week (%)	57.1	65
No dietary restrictions (%)	92.9	85
Any antepartum Probiotics (%)	35.7	15
Gestational Diabetes (%)	0	0
**HOME ENVIRONMENT**		
Bactericidal soap at home (%)	7.1	25
Siblings at home (%)	92.9	50
Furry pets at home (%)	50	70
Rural home environment (%)	50	5
**DELIVERY AND BABY CHARACTERISTICS**		
Baby weight (lbs.) Mean(min-max) SD	7.99 (7-10) .97	7.89 (7-10) .81
Baby sex- female (%)	64	66
Labor duration (minutes) Mean (min-max) SD	599.79 (84-2666) 730.17	664.05 (34-1636)437.38
Rupture of membranes duration (minutes) Mean (min-max) SD	322.57 (5-1672) 503.88	323 (4-1185) 393.75
Exclusive breast feeding first month (%)	100	100
Breastfeeding initiation (minutes after birth) Mean (min/max) SD	47 (9-84) 27	42.24 (15-90) 19
Baby antibiotic eye prophylaxis (%)	0	95
Baby vitamin K injection (%)	64	95
Baby bath first 48 hours (%)	7.1	95
Midwifery care for delivery (%)	100	95
Labor induction, chemical (%)	0	5
Epidural anesthesia (%)	0	5
Labor analgesia (%)	0	10
Episiotomy (%)	0	5
Babies exposed to water birth (%)	28	0
Babies exposed to maternal antibiotics in labor (%)	0	19
Babies exposed to maternal antibiotics in month before or after delivery (%)	0	33

Table S2. Number of samples, sequences, and bacterial OTUs from the samples from infants and mothers in the study

Delivery Site	Home			Hospital			Total
Subject	Baby	Mother	Mother	Baby	Mother	Mother	
Sample type	Feces	Feces	Vagina	Feces	Feces	Vagina	
# Subjects	10	10	10	10	10	10	60
# Samples	61	63	76	57	56	73	386
Total # sequences	1,170,036	2,145,389	1,359,437	1,489,419	2,277,665	1,563,905	10,005,851
Mean # sequences ± Std dev	18,872±8,678	34,053±20,091	17,887±16,589	26,130±13,471	40673±22,215	21,423±17,781	
Total # OTU-yielding sequences	1,156,795	2,066,092	1,340,696	1,475,517	2,201,867	1,547,811	9,788,778
Mean # OTU-yielding sequences ± Std dev	18,657 ± 8,601	32,795 ± 20,353	17,640±16,354	25,886±13,380	39,319±21,468	21,203±17,559	
# OTUs	7,133	22,335	6,830	7,192	21,336	6,669	71,495
Mean # of observed OTUs ± Std dev	375±176	1530± 514	297±230	395±265	1,630±660	302±220	

Table S3. Number of fecal bacterial sequences, and bacterial OTUs from hospital-born babies by antibiotic exposure

Delivery Site	No antibiotics	Intra partum antibiotics	Peripartum antibiotics	Total
Subject	Baby	Baby	Baby	
Sample type	Feces	Feces	Feces	
# Subjects	10	4	7	21
# Samples	57	28	42	127
Total # sequences	1,489,419	521,172	985,358	2,995,949
Mean # sequences + Std dev	26,130 + 13,471	18,613 + 8,605	22,915 + 11,579	
Total # sequences yielding OTUs	1,475,517	517,252	975,148	2,967,917
Mean # sequences yielding OTUs + Std dev	25,886 + 13,380	18,473 + 8,560	22,678 + 11,525	
# Observed OTUs	7,192	8,410	11,025	26,627
Mean # observed OTUs + Std dev	395 + 265	285 + 114	371<br>277	

Table S4. Number of fecal bacterial sequences, and bacterial OTUs from home-born babies (water birth vs. non-water birth)

Delivery Site	Home NSVD	Home NSVD Water	Total
Subject	Baby	Baby	
Sample type	Feces	Feces	
# Subjects	10	4	14
# Samples	61	22	83
Total # sequences	1,170,036	478,818	1,648,854
Mean # of sequences ± Std dev	18,872 ± 8,678	21764 ± 9202	
Total # of sequences yielding OTUs	1,156,795	472,930	1,629,725
Mean # of sequences yielding OTUs ± Std dev	18,657 ± 8,601	21,497 ± 9,060	
# of observed OTUs	7,133	3,622	10,755
Mean # of observed OTUs ± Std dev	375 ± 176	383 ± 166	

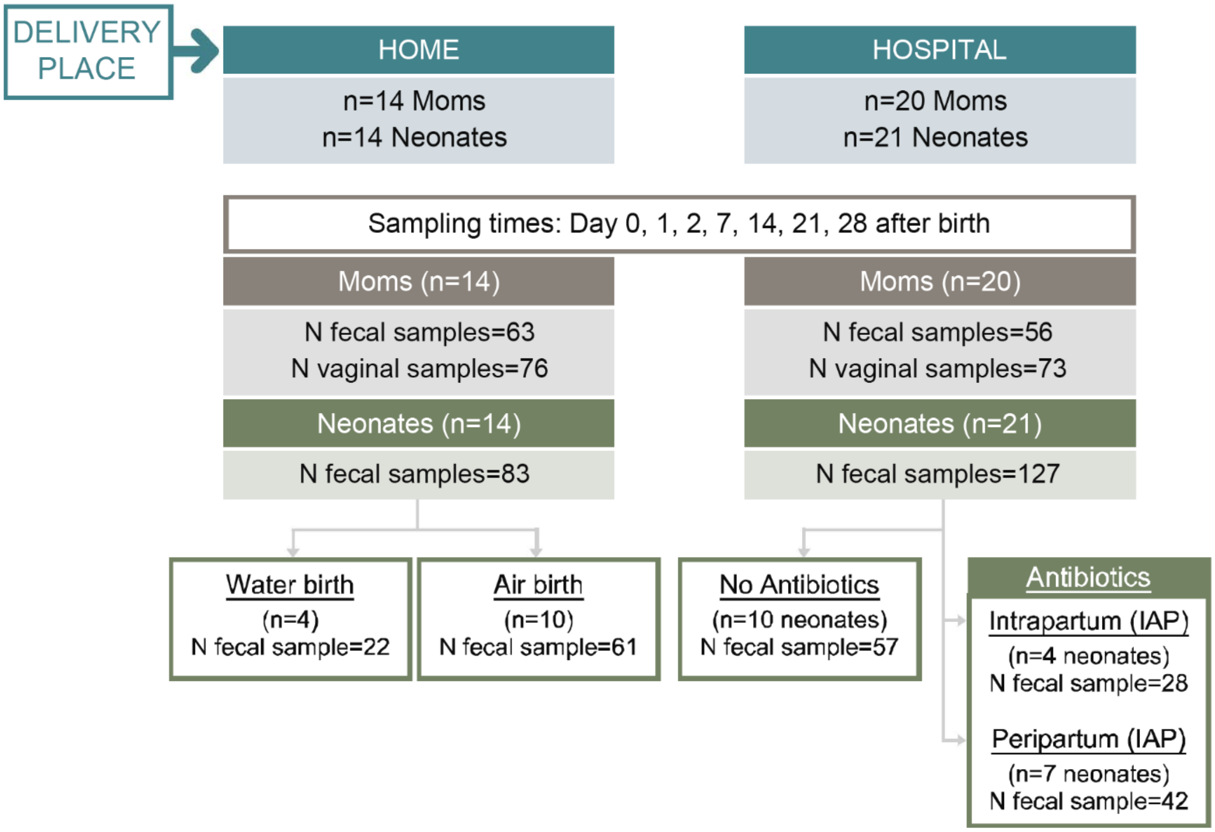

Figure S1. Study design. A total of 34 mothers and their infants, 14 delivered at home and 21 in the hospital were sampled. Of the home-born babies, 4 had water birth, and of the hospital-born babies, 11 were exposed to antibiotics (7 peripartum and 4 intrapartum).

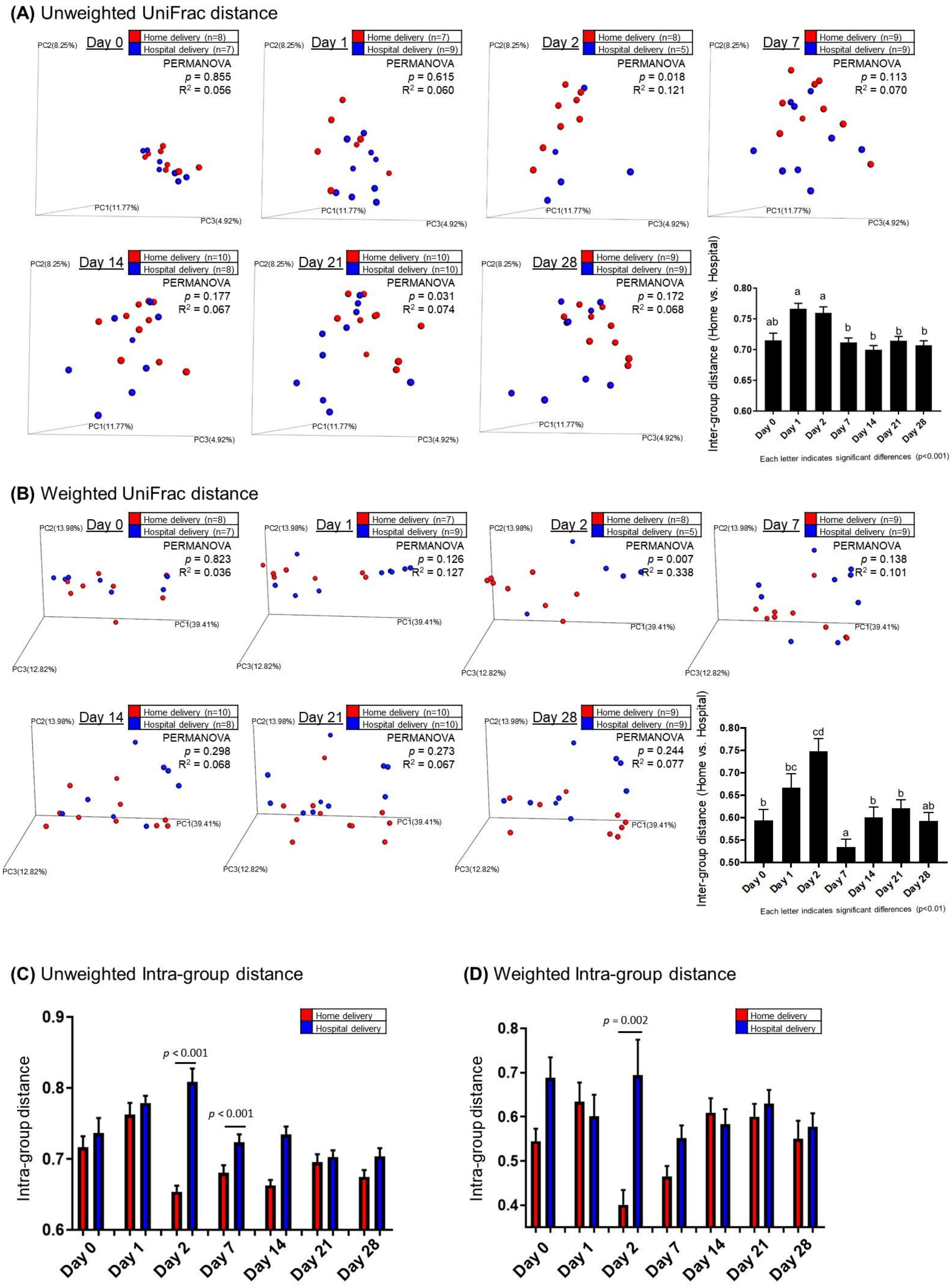

Figure S2. Fecal microbiota diversity in vaginally delivered infants, 10 delivered at home and 10 in the hospital. (A-B) Fecal β -diversity stratified by days after birth. Unweighted **(A)** and weighted **(B)** UniFrac distances. **(C-D)** Box plot of intra-group distances. Unweighted **(C)** and weighted **(D)** UniFrac distances. PERMANOVA was used to test dissimilarity. Non-parametric p value was calculated using 10,000 Monte Carlo permutations. All babies were breastfed and were not exposed to antibiotics.

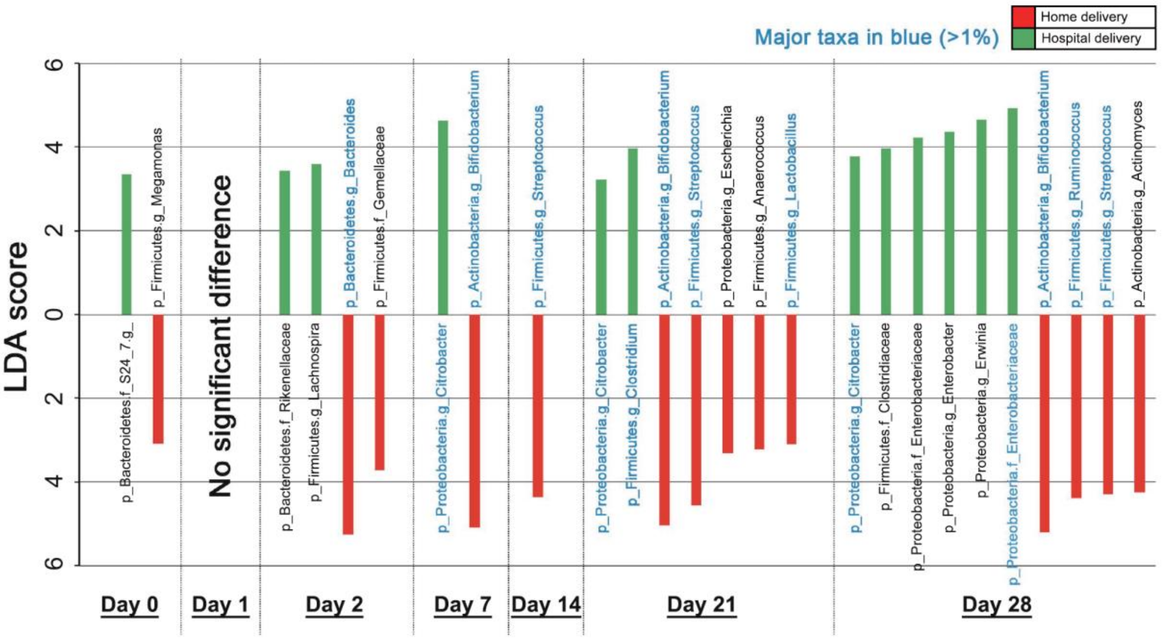

Figure S3. LDA scores of discriminant fecal taxa from infants by birth location. All babies were breastfed and were not exposed to antibiotics.

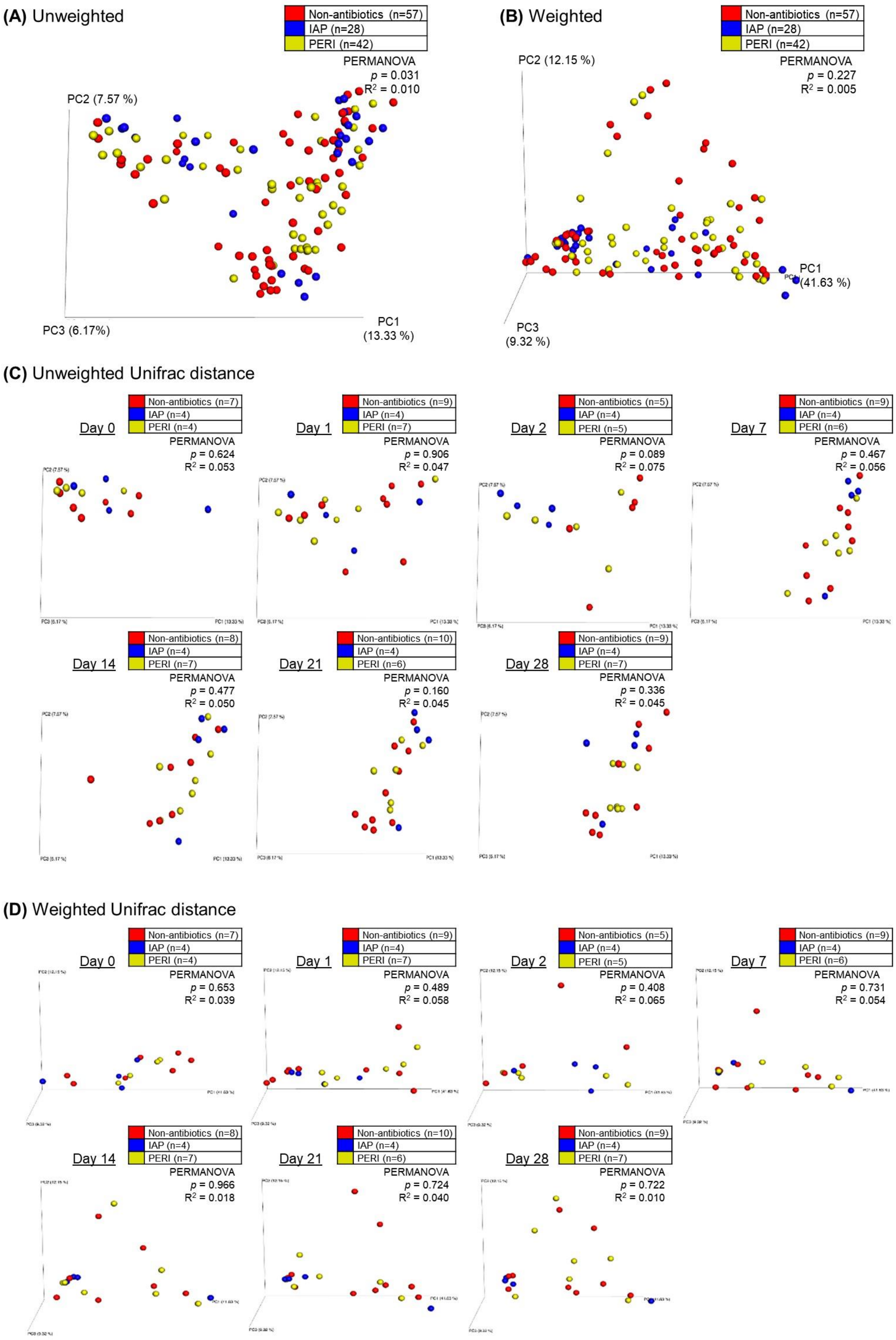

(A) Unweighted
Non-antibiotics (n=57)
IAP (n=28)
PERI (n=42)
PERMANOVA
p = 0.031
R2 = 0.010
PC2 (7.57 %)
PC3 (6.17%)
PC1 (13.33 %)
(B) Weighted
Non-antibiotics (n=57)
IAP (n=28)
PERI (n=42)
PERMANOVA
p = 0.227
R2 = 0.005
PC2 (12.15 %)
PC1 (41.63 %)
PC3 (9.32 %)
(C) Unweighted Unifrac distance
Day 0
Non-antibiotics (n=7)
IAP (n=4)
PERI (n=4)
PERMANOVA
p = 0.624
R2 = 0.053
Day 1
Non-antibiotics (n=9)
IAP (n=4)
PERI (n=7)
PERMANOVA
p = 0.906
R2 = 0.047
Day 2
Non-antibiotics (n=5)
IAP (n=4)
PERI (n=5)
PERMANOVA
p = 0.089
R2 = 0.075
Day 7
Non-antibiotics (n=9)
IAP (n=4)
PERI (n=6)
PERMANOVA
p = 0.467
R2 = 0.056
Day 14
Non-antibiotics (n=8)
IAP (n=4)
PERI (n=7)
PERMANOVA
p = 0.477
R2 = 0.050
Day 21
Non-antibiotics (n=10)
IAP (n=4)
PERI (n=6)
PERMANOVA
p = 0.160
R2 = 0.045
Day 28
Non-antibiotics (n=9)
IAP (n=4)
PERI (n=7)
PERMANOVA
p = 0.336
R2 = 0.045
(D) Weighted Unifrac distance
Day 0
Non-antibiotics (n=7)
IAP (n=4)
PERI (n=4)
PERMANOVA
p = 0.653
R2 = 0.039
Day 1
Non-antibiotics (n=9)
IAP (n=4)
PERI (n=7)
PERMANOVA
p = 0.489
R2 = 0.058
Day 2
Non-antibiotics (n=5)
IAP (n=4)
PERI (n=5)
PERMANOVA
p = 0.408
R2 = 0.065
Day 7
Non-antibiotics (n=9)
IAP (n=4)
PERI (n=6)
PERMANOVA
p = 0.731
R2 = 0.054
Day 14
Non-antibiotics (n=8)
IAP (n=4)
PERI (n=7)
PERMANOVA
p = 0.966
R2 = 0.018
Day 21
Non-antibiotics (n=10)
IAP (n=4)
PERI (n=6)
PERMANOVA
p = 0.724
R2 = 0.040
Day 28
Non-antibiotics (n=9)
IAP (n=4)
PERI (n=7)
PERMANOVA
p = 0.722
R2 = 0.010

Figure S4. Fecal microbiota β -diversity in 21 infants delivered in the hospital, by antibiotic exposure. A total of 11 infants were exposed (4 peripartum and 7 intrapartum IAP). **(A-B)** Fecal β-diversity, unweighted **(A)** and weighted **(B)** UniFrac distances. **(C-D)** Fecal β -diversity stratified by days after birth. PERMANOVA was used to test dissimilarity.

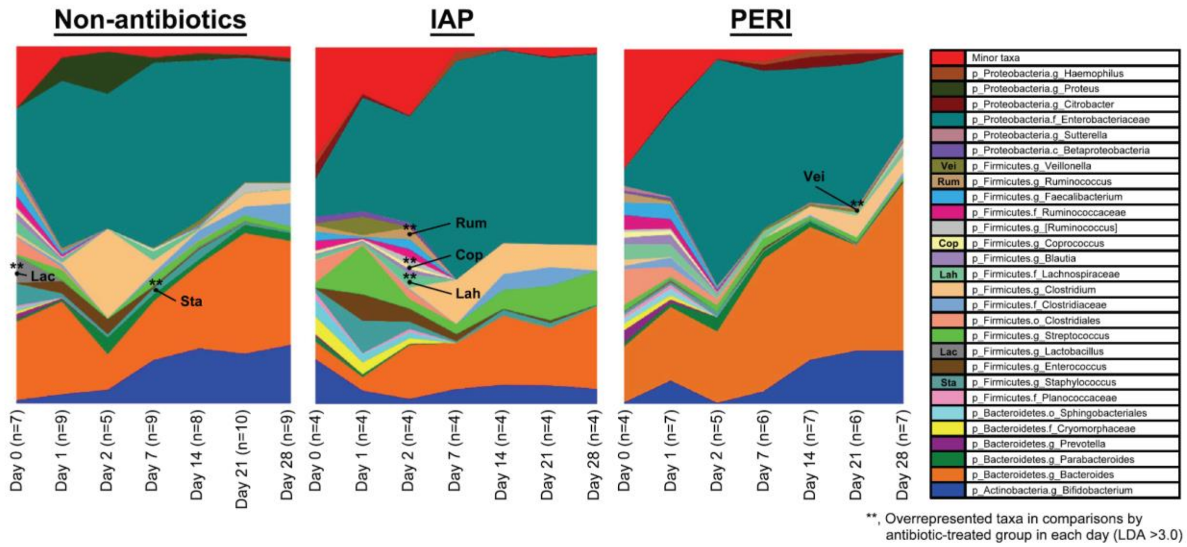

Figure S5. Fecal microbiota β -diversity in 21 infants delivered in the hospital, by antibiotic exposure. A total of 11 infants were exposed (4 peripartum and 7 intrapartum IAP). **, Overrepresented taxa in comparisons by antibiotic-treated group in each day (LDA >3.0).

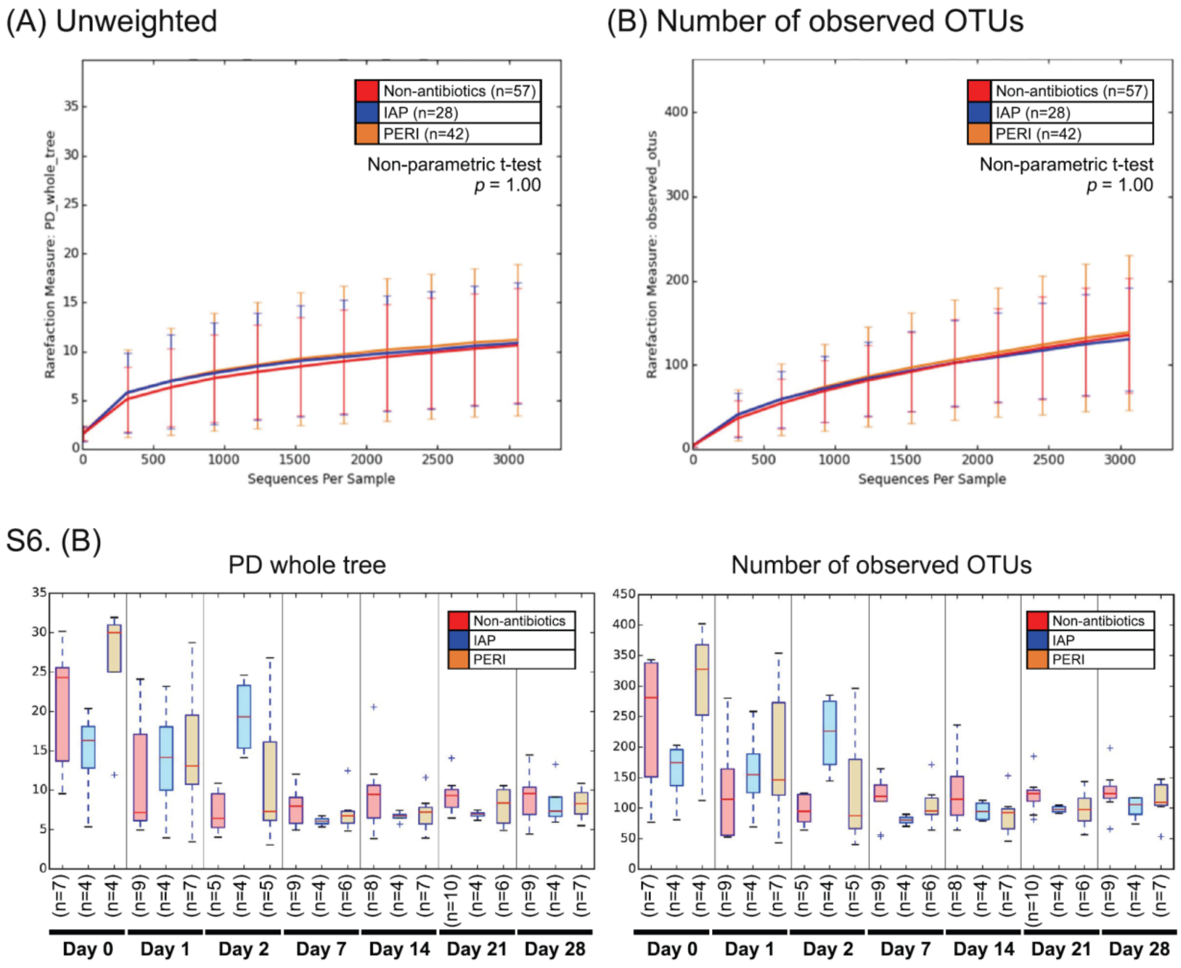

Figure S6. Rarefaction curves of fecal bacteria in 11 infants exposed to antibiotics. A total of 11 infants were exposed (4 peripartum and 7 intrapartum IAP). **(A)** PD whole tree. **(B)** Number of observed OTUs. **(C-D)** Fecal α-diversity stratified by days after birth. PD whole tree **(C)** and number of observed OTUs **(D)**. Non-parametric p value was calculated using 10,000 Monte Carlo permutations.

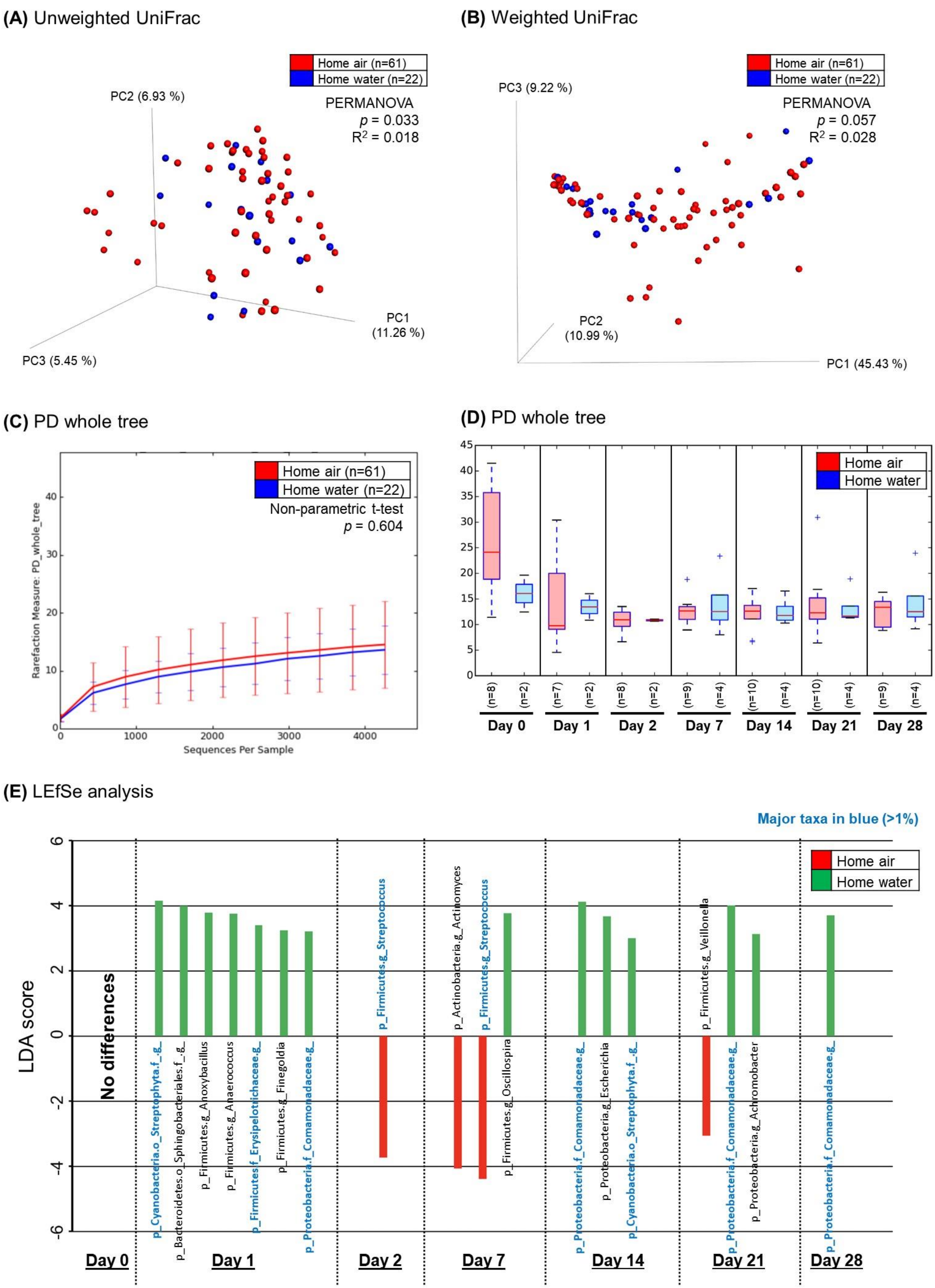
(A) Unweighted UniFrac
Home air (n=61)
Home water (n=22)
PERMANOVA
p = 0.033
R2 = 0.018
PC2 (6.93 %)
PC1 (11.26 %)
PC3 (5.45 %)
(B) Weighted UniFrac
Home air (n=61)
Home water (n=22)
PERMANOVA
p = 0.057
R2 = 0.028
PC3 (9.22 %)
PC2 (10.99 %)
PC1 (45.43 %)
(C) PD whole tree
Home air (n=61)
Home water (n=22)
Non-parametric t-test
p = 0.604
Rarefaction Measure: PD_whole_tree
Sequences Per Sample
0
1000
2000
3000
4000
10
20
30
40
(D) PD whole tree
Home air
Home water
0
5
10
15
20
25
30
35
40
45
(n=8) (n=2) (n=7) (n=2) (n=8) (n=2) (n=9) (n=4) (n=10) (n=4) (n=10) (n=4) (n=9) (n=4)
Day 0
Day 1
Day 2
Day 7
Day 14
Day 21
Day 28
(E) LEfSe analysis
Major taxa in blue (>1%)
Home air
Home water
LDA score
6
4
2
0
-2
-4
-6
No differences
p_Cyanobacteria.o_Streptophyta.f_.g_
p_Bacteroidetes.o_Sphingobacteriales.f_.g_
p_Firmicutes.g_Anoxybacillus
p_Firmicutes.g_Anaerococcus
p_Firmicutes.f_Erysipelotrichaceae.g_
p_Firmicutes.g_Finegoldia
p_Proteobacteria.f_Comamonadaceae.g_
p_Firmicutes.g_Streptococcus
p_Actinobacteria.g_Actinomyces
p_Firmicutes.g_Streptococcus
p_Firmicutes.g_Oscillospira
p_Proteobacteria.f_Comamonadaceae.g_
p_Proteobacteria.g_Escherichia
p_Cyanobacteria.o_Streptophyta.f_.g_
p_Firmicutes.g_Veillonella
p_Proteobacteria.f_Comamonadaceae.g_
p_Proteobacteria.g_Achromobacter
p_Proteobacteria.f_Comamonadaceae.g_
Day 0
Day 1
Day 2
Day 7
Day 14
Day 21
Day 28

Figure S7. Fecal microbiota diversity in 14 home-born infants, of which 4 were delivered in water, and 10 were delivered in air. (A-B) Fecal β–diversity unweighted **(A)** and weighted **(B)** UniFrac distances. **(C)** α-diversity (PD whole tree) of fecal microbiota. Non-parametric p value was calculated using 10,000 Monte Carlo permutations. **(D)** α-diversity of fecal microbiota stratified by days after birth. **(E)** LDA scores of discriminant fecal taxa from home-born infants.

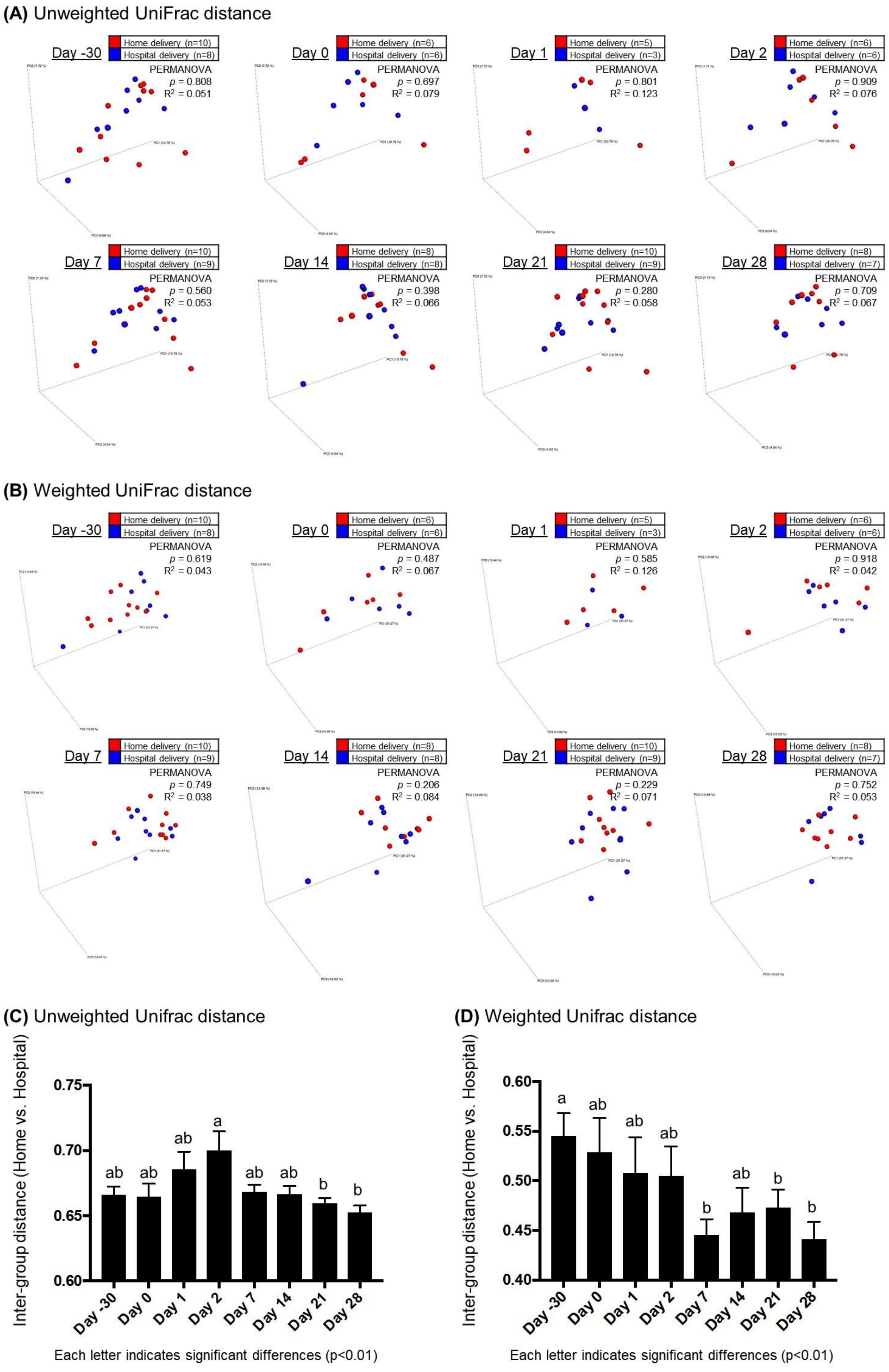
(A) Unweighted UniFrac distance
Day -30
Home delivery (n=10)
Hospital delivery (n=8)
PERMANOVA
p = 0.808
R² = 0.051
Day 0
Home delivery (n=6)
Hospital delivery (n=6)
PERMANOVA
p = 0.697
R² = 0.079
Day 1
Home delivery (n=5)
Hospital delivery (n=3)
PERMANOVA
p = 0.801
R² = 0.123
Day 2
Home delivery (n=6)
Hospital delivery (n=6)
PERMANOVA
p = 0.909
R² = 0.076
Day 7
Home delivery (n=10)
Hospital delivery (n=9)
PERMANOVA
p = 0.560
R² = 0.053
Day 14
Home delivery (n=8)
Hospital delivery (n=8)
PERMANOVA
p = 0.398
R² = 0.066
Day 21
Home delivery (n=10)
Hospital delivery (n=9)
PERMANOVA
p = 0.280
R² = 0.058
Day 28
Home delivery (n=8)
Hospital delivery (n=7)
PERMANOVA
p = 0.709
R² = 0.067
(B) Weighted UniFrac distance
Day -30
Home delivery (n=10)
Hospital delivery (n=8)
PERMANOVA
p = 0.619
R² = 0.043
Day 0
Home delivery (n=6)
Hospital delivery (n=6)
PERMANOVA
p = 0.487
R² = 0.067
Day 1
Home delivery (n=5)
Hospital delivery (n=3)
PERMANOVA
p = 0.585
R² = 0.126
Day 2
Home delivery (n=6)
Hospital delivery (n=6)
PERMANOVA
p = 0.918
R² = 0.042
Day 7
Home delivery (n=10)
Hospital delivery (n=9)
PERMANOVA
p = 0.749
R² = 0.038
Day 14
Home delivery (n=8)
Hospital delivery (n=8)
PERMANOVA
p = 0.206
R² = 0.084
Day 21
Home delivery (n=10)
Hospital delivery (n=9)
PERMANOVA
p = 0.229
R² = 0.071
Day 28
Home delivery (n=8)
Hospital delivery (n=7)
PERMANOVA
p = 0.752
R² = 0.053
(C) Unweighted Unifrac distance
Inter-group distance (Home vs. Hospital)
0.75
0.70
0.65
0.60
ab
ab
ab
a
ab
ab
b
b
Day -30
Day 0
Day 1
Day 2
Day 7
Day 14
Day 21
Day 28
Each letter indicates significant differences (p<0.01)
(D) Weighted Unifrac distance
Inter-group distance (Home vs. Hospital)
0.60
0.55
0.50
0.45
0.40
a
ab
ab
ab
b
ab
b
b
Day -30
Day 0
Day 1
Day 2
Day 7
Day 14
Day 21
Day 28
Each letter indicates significant differences (p<0.01)

Figure S8. Fecal β-diversity in 20 mothers who delivered vaginally, 10 at home and 10 in the hospital. (A-B) Fecal β-diversity stratified by days after birth. Unweighted **(A)** and weighted **(B)** UniFrac distances. **(C-D)** Box plot of inter-group unweighted **(C)** and weighted **(D)** UniFrac distances.

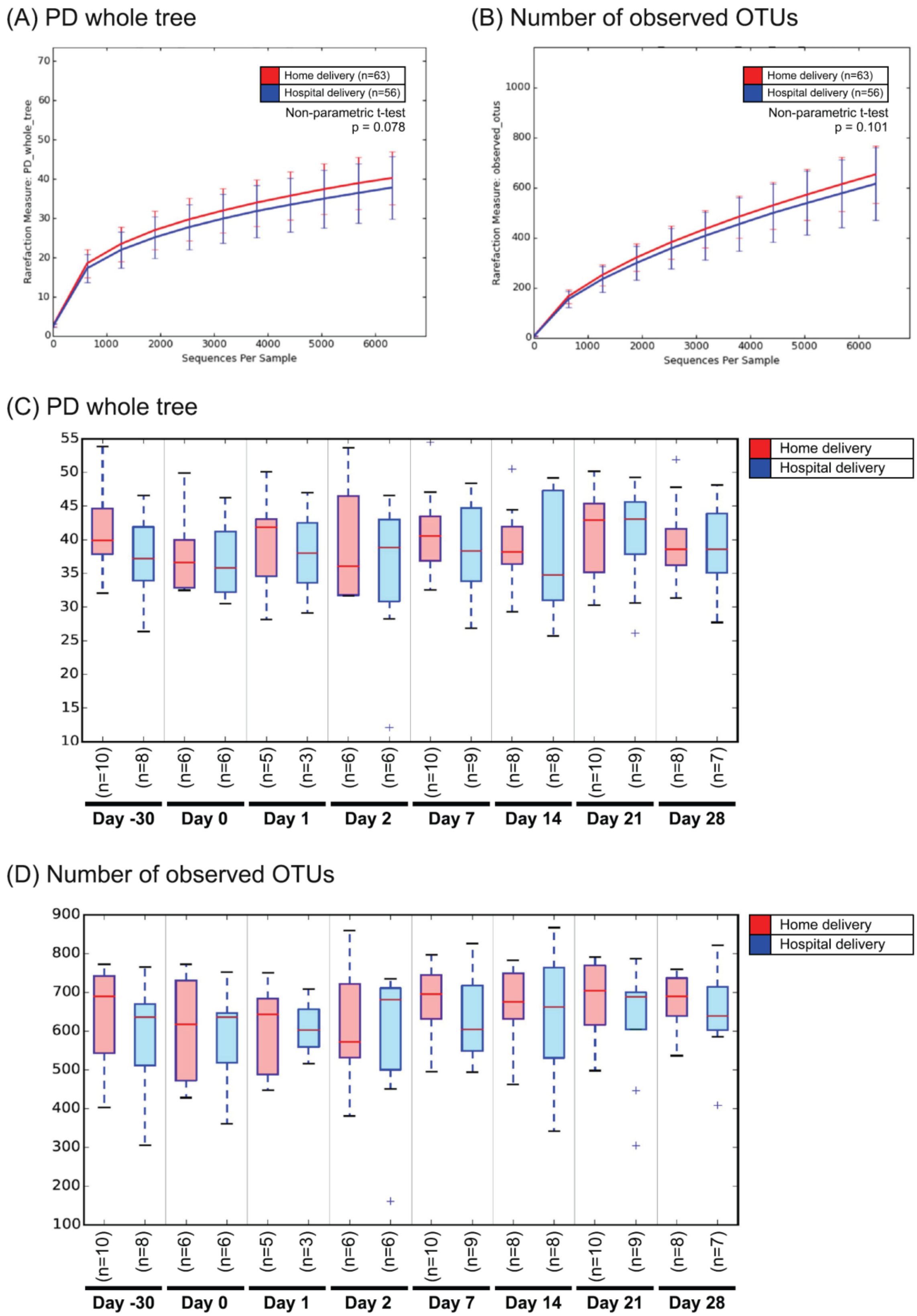

Figure S9. Fecal α-diversity in 20 mothers who delivered vaginally, 10 at home and 10 in the hospital. (A-B) α-diversity of fecal microbiota. α-diversity PD whole tree **(A)** and number of observed OTUs **(B)**. **(C-D) α-diversity of fecal microbiota stratified by days after birth.** α-diversity PD whole tree **(C)** and number of observed OTUs **(D)**. Non-parametric p value was calculated using 10,000 Monte Carlo permutations.

(A)

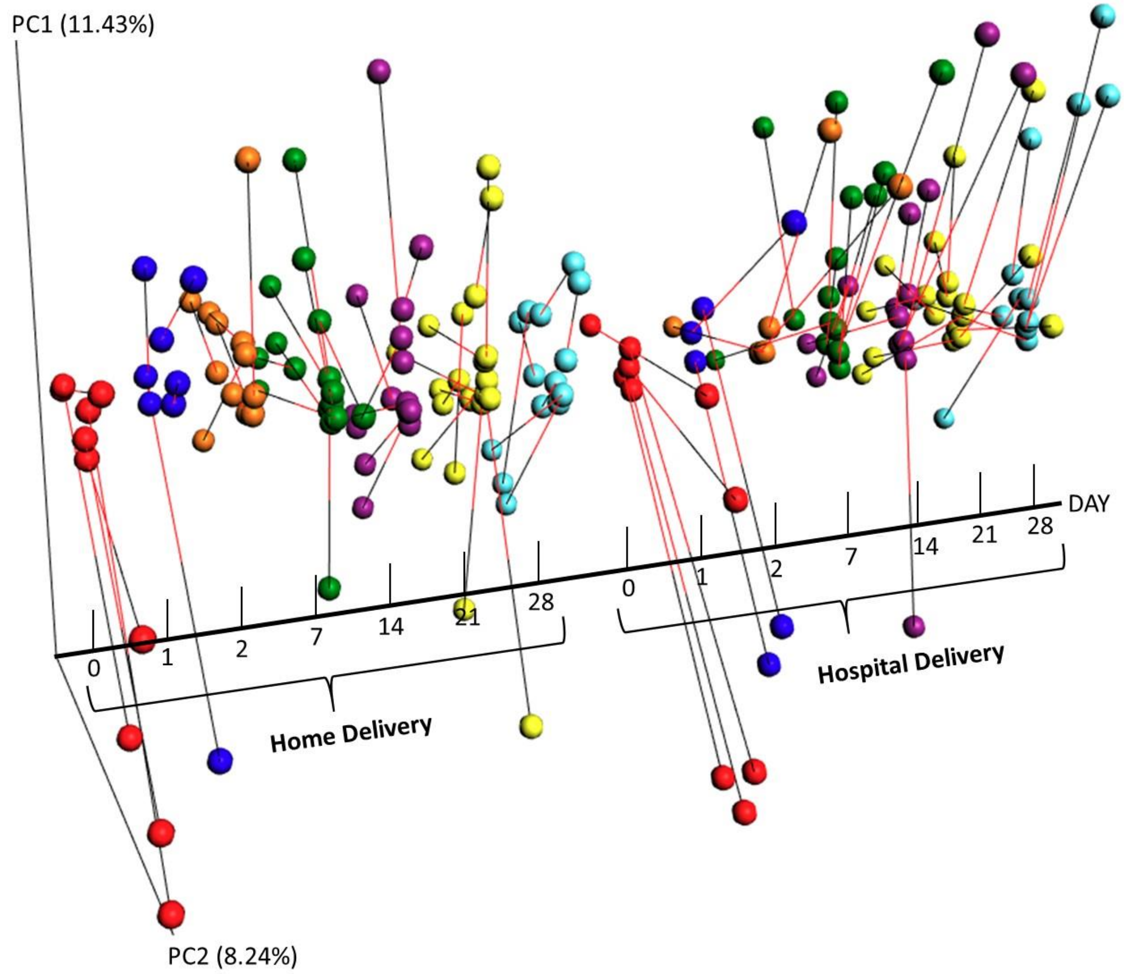

(B)

	Sampling Day	Day0	Day01	Day02	Day07	Day14	Day21	Day28
**Home Delivery**	**Average**	0.0665	0.0074	0.0200	0.0692	0.0065	0.0197	0.0047
	STDEV	0.0768	0.0050	0.0275	0.1314	0.0033	0.0470	0.0024
**Hospital Delivery**	**Average**	0.2700	0.0782	0.0295	0.0394	0.1480	0.0116	0.0351
	STDEV	0.2841	0.1030	0.0404	0.0964	0.2398	0.0207	0.0799
	p-value	0.2040	0.2981	0.7238	0.6267	0.1443	0.6585	0.3711

Figure S10. Comparing fecal microbiotas from mother with their own babies. (A) Procrustes plot comparing principal coordinates of unweighted UniFrac distances, from the same dyad of the mothers (connected by the black line) with their babies (connected by the red line). Each color indicates sampling day. **(B)** Proportion of detected mother's fecal sample-derived OTUs in their baby's fecal samples using Source tracking analysis. Student t-test was used for statistical test.

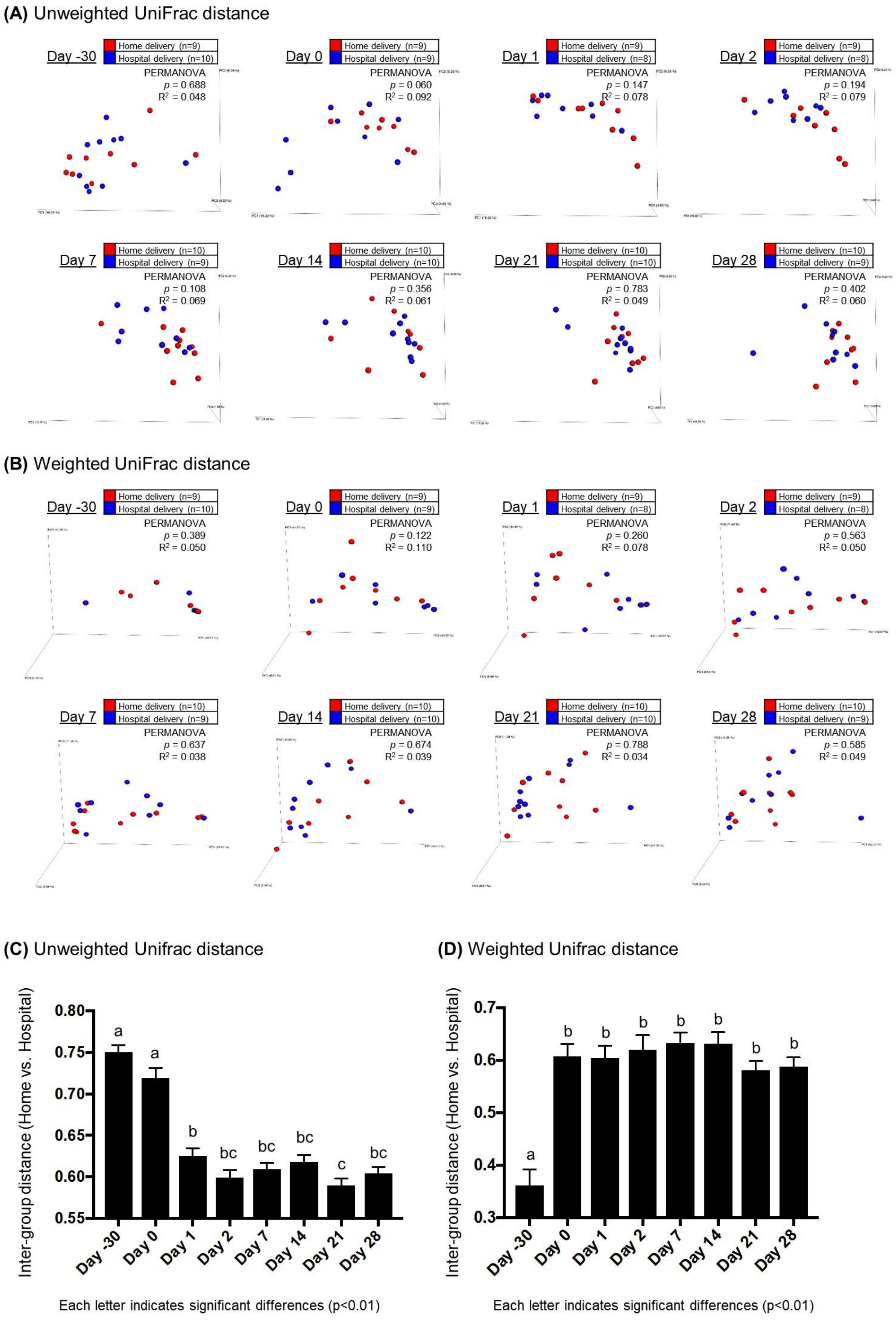
(A) Unweighted UniFrac distance
Day -30
Home delivery (n=9)
Hospital delivery (n=10)
PERMANOVA
p = 0.688
R2 = 0.048
Day 0
Home delivery (n=9)
Hospital delivery (n=9)
PERMANOVA
p = 0.060
R2 = 0.092
Day 1
Home delivery (n=9)
Hospital delivery (n=8)
PERMANOVA
p = 0.147
R2 = 0.078
Day 2
Home delivery (n=9)
Hospital delivery (n=8)
PERMANOVA
p = 0.194
R2 = 0.079
Day 7
Home delivery (n=10)
Hospital delivery (n=9)
PERMANOVA
p = 0.108
R2 = 0.069
Day 14
Home delivery (n=10)
Hospital delivery (n=10)
PERMANOVA
p = 0.356
R2 = 0.061
Day 21
Home delivery (n=10)
Hospital delivery (n=10)
PERMANOVA
p = 0.783
R2 = 0.049
Day 28
Home delivery (n=10)
Hospital delivery (n=9)
PERMANOVA
p = 0.402
R2 = 0.060
(B) Weighted UniFrac distance
Day -30
Home delivery (n=9)
Hospital delivery (n=10)
PERMANOVA
p = 0.389
R2 = 0.050
Day 0
Home delivery (n=9)
Hospital delivery (n=9)
PERMANOVA
p = 0.122
R2 = 0.110
Day 1
Home delivery (n=9)
Hospital delivery (n=8)
PERMANOVA
p = 0.260
R2 = 0.078
Day 2
Home delivery (n=9)
Hospital delivery (n=8)
PERMANOVA
p = 0.563
R2 = 0.050
Day 7
Home delivery (n=10)
Hospital delivery (n=9)
PERMANOVA
p = 0.637
R2 = 0.038
Day 14
Home delivery (n=10)
Hospital delivery (n=10)
PERMANOVA
p = 0.674
R2 = 0.039
Day 21
Home delivery (n=10)
Hospital delivery (n=10)
PERMANOVA
p = 0.788
R2 = 0.034
Day 28
Home delivery (n=10)
Hospital delivery (n=9)
PERMANOVA
p = 0.585
R2 = 0.049
(C) Unweighted Unifrac distance
Inter-group distance (Home vs. Hospital)
0.80
0.75
0.70
0.65
0.60
0.55
a
a
b
bc
bc
bc
c
bc
Day -30
Day 0
Day 1
Day 2
Day 7
Day 14
Day 21
Day 28
Each letter indicates significant differences (p<0.01)
(D) Weighted Unifrac distance
Inter-group distance (Home vs. Hospital)
0.7
0.6
0.5
0.4
0.3
a
b
b
b
b
b
b
b
Day -30
Day 0
Day 1
Day 2
Day 7
Day 14
Day 21
Day 28
Each letter indicates significant differences (p<0.01)

Figure S11. Vaginal β-diversity in 20 mothers who delivered vaginally, 10 at home and 10 in the hospital. (A-B) Vaginal β-diversity stratified by days after birth, unweighted **(A)** and weighted **(B)** UniFrac distances. **(C-D)** Box plot of inter-group unweighted **(C)** and weighted **(D)** UniFrac distances. Non-parametric p value was calculated using 10,000 Monte Carlo permutations. Each letter indicates significant differences ($p<0.01$).

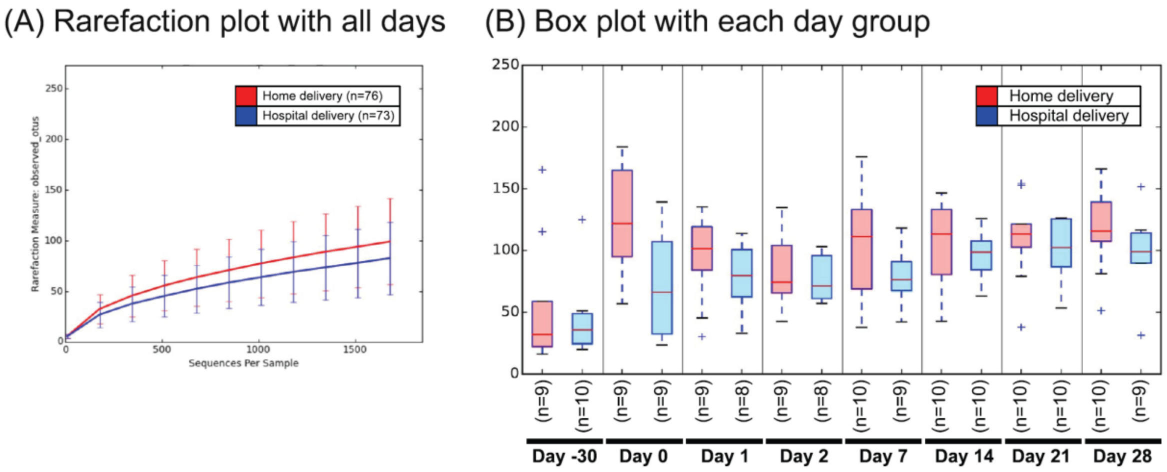

Figure S12. Vaginal α-diversity in 20 mothers who delivered vaginally, 10 at home and 10 in the hospital. (A) α-diversity observed OTUs. Non-parametric p value was calculated using 10,000 Monte Carlo permutation. **(B)** α-diversity observed OTUs stratified by days after birth.

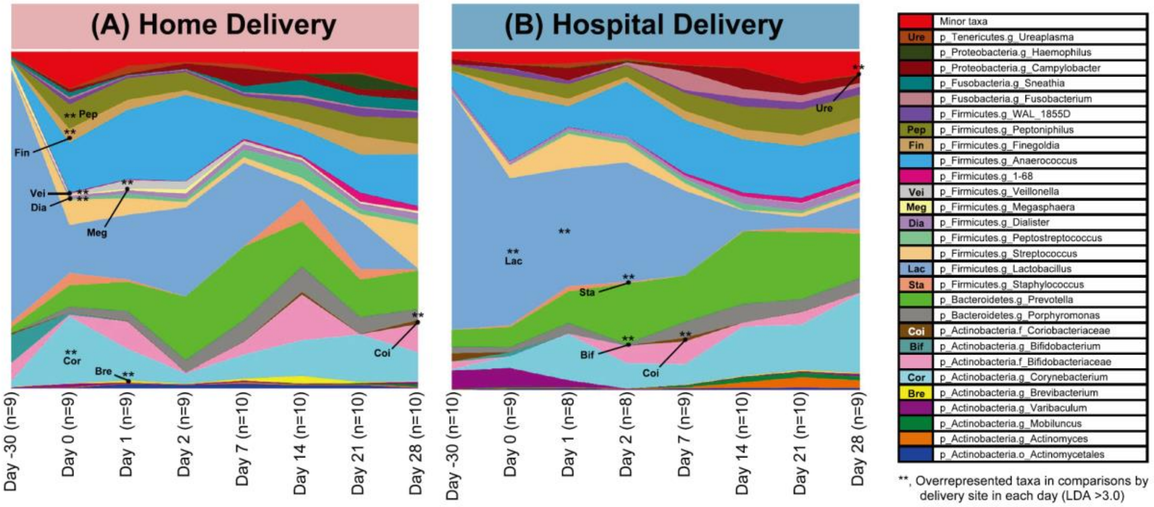

Figure S13. Taxa plots of vaginal microbiota in 20 mothers who delivered vaginally, 10 at home (A) and 10 in the hospital (B). ** Overrepresented taxa in comparisons by delivery site group in each day (LDA >3.0).

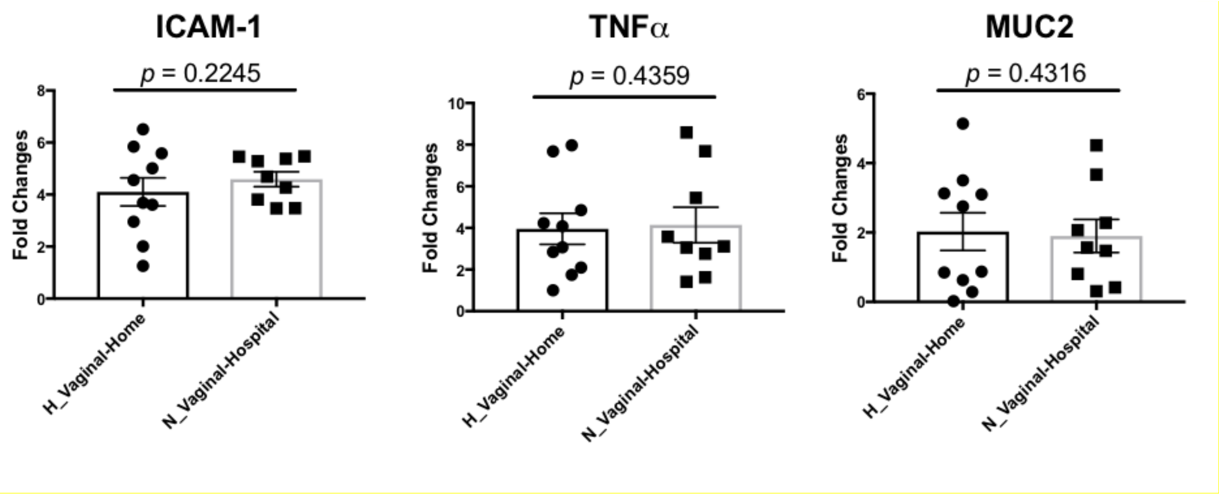

Figure S14. Human colonic epithelial HT-29 cells gene (ICAM-1, TNFα, and MUC2) expression following exposure to sterile fecal water generated from 1 month old infant feces delivered in a home (n=10) or hospital environment (n=9). Gene expression of a subset of epithelial markers differs by home or hospital birth. Mean ± SEM are shown.
